# The case of the neonate vs. LMIC medical academia—a jury-style systematic review of 32 years of literature without significant mortality reduction

**DOI:** 10.3389/fped.2024.1413113

**Published:** 2024-07-22

**Authors:** Hippolite O. Amadi, Ifeoluwa O. Abioye, Ukadike C. Ugbolue, Rhoda-Dara Ekpenyong, Nnamdi F. Ekwem, Ogechi J. Nwaneri, Chidiebere Dike

**Affiliations:** ^1^ Department of Bioengineering, Imperial College London, London, United Kingdom; ^2^ Division of Precision Health, Quality and Safety Leadership, University of Calgary, Alberta, AB, Canada; ^3^ Biomechanics Laboratory, Division of Sports and Exercise, University of the West of Scotland, Glasgow, United Kingdom; ^4^ Department of Paediatrics, University of Calabar, Calabar, Nigeria; ^5^ Corporate & Commercial Law, Frederick & Co., Barristers and Associates, Abuja, Nigeria; ^6^ Division of Nursing Education, School of Nursing & Midwifery, Mount Royal University, Alberta, AB, Canada; ^7^ Health and Education Department, Manchester Metropolitan University, Manchester, United Kingdom

**Keywords:** Nigerian neonate, neonatal mortality, preterm neonate, low birth weight, first 7 days of life

## Abstract

**Introduction:**

The high neonatal mortality rate in low- and middle-income countries (LMICs) such as Nigeria has lasted for more than 30 years to date with associated nursing fatigue. Despite prominent hard work, technological improvements, and many publications released from the country since 1990, the problem has persisted, perhaps due to a lack of intervention scale-up. Could there be neglected discoveries unwittingly abandoned by Nigerian policymakers over the years, perhaps locked up in previous publications? A careful review may reveal these insights to alert policymakers, inspire researchers, and refocus in-country research efforts towards impactful directions for improving neonatal survival rates. The focus was to determine the prevailed effectiveness of LMIC medical academia in creating solutions to end the high neonatal mortality rate.

**Methods:**

An unconventional systematic review protocol structure following the PRISMA 2020 checklist was designed and registered at INPLASY (registration number: INPLASY202380096, doi: 10.37766/inplasy2023.8.0096). A jury of paediatricians was assembled and observed by a team of legal professionals. The jury searched the literature from 1990 to the end of 2022, extracted newborn-related articles about Nigeria, and assessed and debated them against expected criteria for solution creation, translation, scale-up, sustainability, and national coverage. Each juror used preset criteria to produce a verdict on the possibility of a published novel idea being a potential game-changer for improving the survival rate of Nigerian neonates.

**Results:**

A summation of the results showed that 19 out of 4,286 publications were assessed to possess potential strategies or interventions to reduce neonatal mortality. Fourteen were fully developed but not appropriately scaled up across the country, hence denying neonates proper access to these interventions.

**Conclusion:**

Nigeria may already have the required game-changing ideas to strategically scale up across the nation to accelerate neonatal survival. Therefore, LMIC healthcare systems may have to look inward to strengthen what they already possess.

**Systematic Review Registration:**

https://inplasy.com/, identifier (INPLASY202380096).

## Introduction

Medical academia in low- and middle-income countries (LMICs) possess the advantage of a better knowledge of the challenges that affect healthcare in their settings. These challenges could be sociocultural, infrastructural, and political factors that could easily be hidden from international agencies that support policy implementations in their countries. Therefore, the duties of LMIC medical academia, such as conducting research and creating solutions for local scientific needs, must never be neglected, irrespective of the volume of imported ideas into their countries. A lack of an active forefront role for LMIC academia could be a major limiting factor in creating sustainable solutions to reduce neonatal mortality rates (NMRs) in LMICs.

Since the 1990s, there have been concerted efforts in LMICs, such as Nigeria, to seek and implement pathways for reducing NMR. Generations of hardworking Nigerian academics have used whatever was available to them to make improvements. However, recent demographic reports—especially following the verdicts of Millennium Development Goal 4 (MDG4)—still suggest that Nigeria has made no significant progress towards NMR reduction. With an estimated population of 223 million people, Nigeria experiences a daily newborn death rate of 846 (1)—the highest in the world. It is widely agreed that many Nigerian neonates still die of preventable causes, with neonatal interventions still largely reserved for the few in major cities where most hospitals with neonatal care units are located (2). Neonates remain the most vulnerable population with limited advocacy for their right to life and access to potential game-changing applications for “neonatal death prevention” in the Nigerian context. The continuing failure of the Nigerian system to protect neonates seems to have become a norm, a huge source of nursing fatigue, and an unwelcome situation for which no one is held accountable. However, it is yet to be understood whether the lack of decisive solutions for this neonatal failure is due to a lack of understanding, poor research techniques, or academic weakness on the part of the Nigerian medical academia, whose duty it is to synthesise the required solutions, or whether the fault lies in the failures of the Federal Ministry of Health (FMOH) of Nigeria to support and inspire indigenous medical research. It is necessary to assess what mitigants the medical academics have provided—has the research strategy towards under-five (U5) mortality reduction been wrong, or the academia been misfiring at the wrong target? Has the medical academia been poor in tactics, neglecting to target the most vulnerable aspects of the U5 lifespan, where it mattered most? Have donor/funding agencies and the FMOH been funding/supporting the wrong research collaborations, leading to 30 years of “insignificant progress”? The questions about this failure and the probable restoration of neonatal hope may only be achieved by identifying the wrong steps of the custodians of Nigeria's neonatal health and proffering suggestions that could reposition the drive for effectively eliminating preventable neonatal deaths in Nigeria. The Nigerian neonate may have been treated unfairly, and there is an urgent need to test the case of the “Nigerian neonate (plaintiff) vs. Nigerian medical academia (defendants) along with FMOH and their leadership appointees at the tertiary hospitals” to verify why academic efforts have not done enough to significantly lower the neonatal mortality rate since the 1990s (3). Nigeria, in this context, is an example case study, as similar situations are faced by many other LMICs who are currently struggling to reduce the high mortality and morbidity rates.

### What is already known

**Figure 1:**
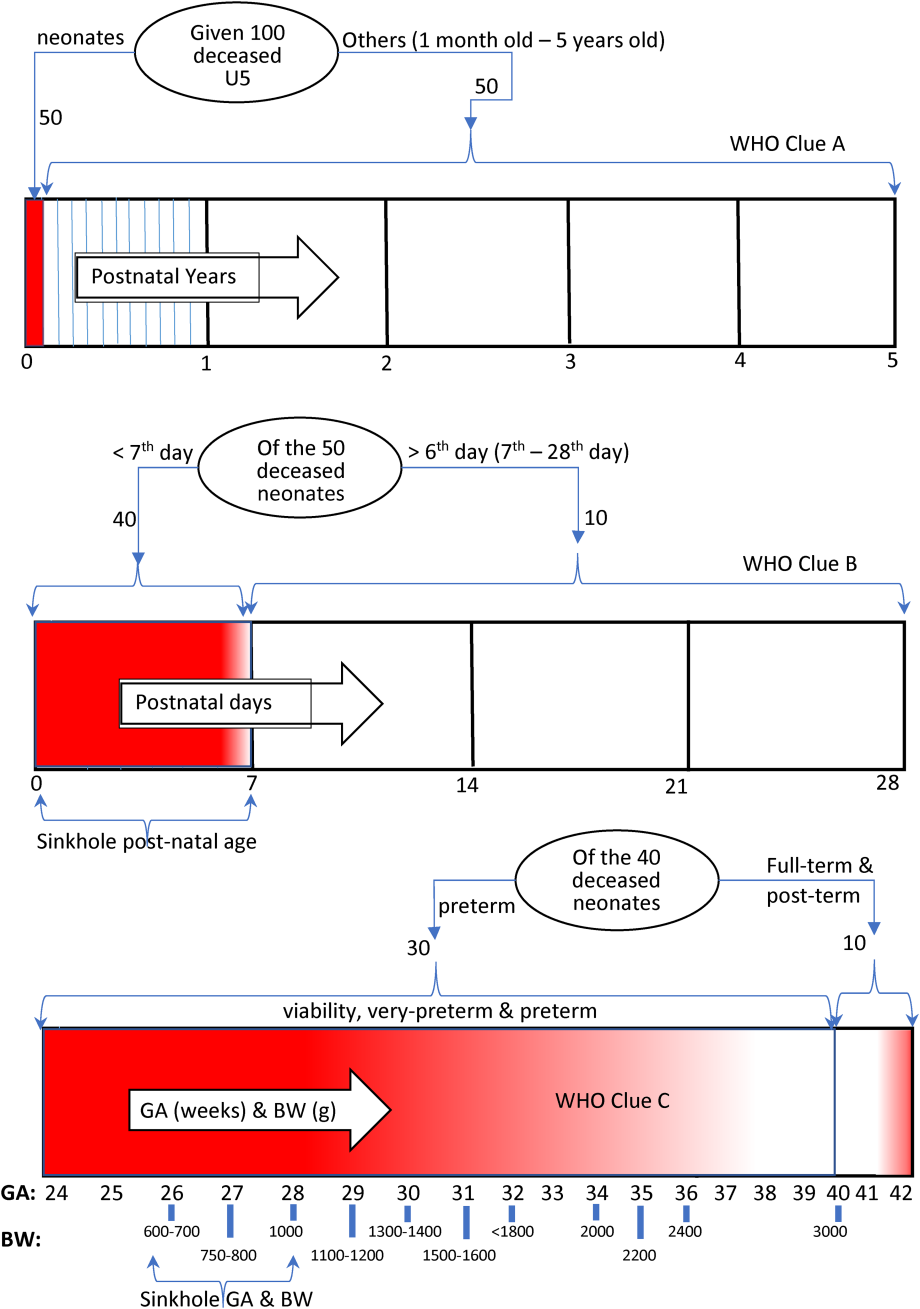
Depiction of mortality burden concentration in U5 lifespan—higher (red) and lower (white). GA, gestational age in weeks; BW, birth weight in grams.

Clue A: Various publications by the World Health Organization (WHO) and United Nations Children's Fund (UNICEF) during the last 10 years of the UN's MDG4 indicated that nearly 50% of under-5 years mortality in Nigeria were neonatal deaths (4), which highlighted the neonatal age as the most devastated population group to focus on to achieve a reduction in under-5 mortality (4, 5). Clue B: Within the first 28 days of neonatal life, the literature identifies the first 7 days (F7D) as the period during which four out of five neonatal mortalities in Nigeria occur (5, 6). Hence, there might be no interventions that could significantly reduce neonatal mortality without, first, successfully addressing and eliminating preventable deaths during F7D. Clue C: The literature further identified that perinatal asphyxia, infection, and prematurity were collectively responsible for nearly 83% of the neonatal deaths in Nigeria (7). However, prematurity was an important risk factor, with 75%–80% of these neonatal deaths occurring among preterm and low-birth-weight neonates, irrespective of the dominant cause of death (8). These three clues are diagrammatically demonstrated in Figure 1, clearly showing that the weakest points with the higher concentration of deaths in the U5 lifespan are fully identifiable—here referred to as the “sinkhole” (in red). This suggests that the target for any game change should be solving the sinkhole inadequacies of the F7D period. The sinkhole casualties are widened by the high incidence rate of “intrauterine growth-retarded” neonates, resulting in abnormally low birth weight for gestational age. In addition, sinkhole casualties are more prevalent in rural poorer communities (9). Hence, targeted low-cost applications for treating such tiny neonates are required to be developed to prevent a high death rate. Deaths at sinkhole points, which are common in many LMICs, could be prevented by providing temperature stability, respiratory support, and treatments for infections, neonatal jaundice, and hypoglycaemia (10). However, most conventional technologies for achieving these tasks are designed for high-income countries (HICs) and are either unaffordable or unsuitable in LMIC settings (10). It is expected that LMIC-specific solutions must have been researched over the years by the LMIC academia, targeting these sinkholes to consequently pave the way for a drastic reduction in U5 mortality. However, if such deliberate efforts have not been actualised, this may hold answers for Nigeria's never-ending high NMR, for which there is a need to identify the liable wrongdoers and proffer recommendations for effective neonatal care strategies.

We seek to verify whether this is a case of academia's misjudgement of the real targets—challenges of the sinkhole casualties during the F7D period—which ought to have been discovered and rendered impotent. Therefore, the objectives of this investigation were to (1) systematically search the literature and ascertain if there had been substantial Nigerian studies that proffered some viable solutions specifically targeting preterm neonates in the F7D of life, whether Nigeria captured such messages and ideas, and perhaps, why such ideas were not scaled up; and (2) promote a mixed methods approach designed to evaluate game-changing technologies or solutions targeting the most vulnerable neonates at age F7D.

## Materials and methods

A systematic review protocol structure following the PRISMA 2020 checklist was designed and registered at INPLASY (registration number: INPLASY202380096, doi: 10.37766/inplasy2023.8.0096) as a standard for this investigation. Hence, the unconventional jury panel technique was deployed to investigate why Nigerian neonates still die in huge numbers. At conceptualisation, the inquest was themed “The Case of the Nigerian Neonate vs. The Nigerian Medical Academia (NMA) & Ors” within the jurisdiction of the Nigerian intelligentsia, made up of young Nigerian paediatricians. The Nigerian neonate was identified as the ultimate victim of the 32 years in question, but the intelligentsia was to determine who was at fault for this. The main perpetrator could be the NMA, but other influencing bodies were to be investigated as well—such as the FMOH, hospital management, WHO, and UNICEF, as these are alleged wrongdoers who may have wittingly or unwittingly contributed to the plight of the Nigerian neonate by failing to warn the FMOH against their wrong directions and strategies. If found liable, the intelligentsia would determine to what extent each party is responsible.

In a typical jury setting, a set number of randomly picked citizens (jurors) are selected to assist in deciding a case. The presiding judge—chief arbiter—explains the case to the jurors, provides evidence and clues, guides them, and specifies the dilemmas of the case that the jurors would investigate as a team using the provided evidence. It is the duty of the judge to ensure that the jurors understand the case being tried and what constitutes an offence; hence, the judge could summon the jurors for briefing until there is a conviction that the jurors have understood the essentials of the wrongdoing. The judge may extend deliberation to enable the jury to reach the required unanimity or supermajority in their verdict to avoid a deadlock. The jurors would discuss, argue, and vote on the case to return a “liable” or “not liable” verdict. Inspired by the jury system in the present inquest, four passionate young Nigerian paediatricians were recruited to form the jury panel as jurors. A benchmark of experience requirements was set as a guide for the recruitment of the jurors, which, amongst others, included the following criteria: (1) must have >5 and <15 years of post-qualification experience as a doctor, (2) must have continuously practiced in neonatology for a minimum of 3 years within this period, (3) must be a qualified consultant, or in the part-2 (final) stage of consultant qualification training, or >5 years working as a senior medical officer in newborn care, and (4) must have achieved research co-authorship in >3 published journal articles. A relatively more senior and well-experienced researcher served as the arbiter, assisted by another senior researcher who chaired the hearing sessions during discussions of issues of conflicting interest with the primary arbiter. A guest arbiter, a senior nursing fellow, was recruited to stand in during the unlikely event of the absence of the assistant arbiter in any session. The arbiters were chosen from a wider medical spectrum of highly experienced scientific researchers with >10 years of research leadership experience, holding ranks from associate to full professors, or senior professional qualifications such as “RN” with over 15 years of experience. The third group in the setup was the observers. This group comprised two practicing lawyers of judicial competence who were able to attend the jury sittings to observe the fairness of the debates and decisions.

### Considerations of conflicts of interest

As a necessity, all the constituent parties in the investigation panel—arbiters, jurors (paediatricians), and observers—were screened to minimise the possibilities of conflicts of interest. All confirmed the independence of their opinions and declared their ability to maintain unbiased opinions. The arbiters interviewed and selected the jurors from early-career practicing paediatricians in Nigeria, who do not have any baggage of personal guilt towards the neonatal failure on trial.

### Systematic review

A possibility might exist where previous publications have provided answers to the current neonatal dilemmas but have been swallowed up in piles of unutilised findings in the last 32 years. It would be unfair to assume that the hardworking Nigeria academia and research community did not provide answers. Typically, a systematic review meticulously delivers a summary of all available primary research relating to a specific research question. Therefore, the systematic review technique, albeit modified, was used to carefully assess the existing literature for the country and provide unbiased recommendations for the LMIC context.

### Search strategy

In our modified adjudication panel style, the literature was assessed on titles addressing Nigerian U5, infant, and neonatal mortality and morbidity from 1990 to 2022. The local research efficiency of NMA was investigated, essentially for the challenges during the F7D period of neonatal life. Therefore, the search specifically looked for studies highlighting new solutions to existing Nigerian problems—research conducted within Nigeria, rather than global initiatives. The arbiters scoped the literature on titles that addressed “Nigeria and under-five,” “Nigeria and infant or infants,” and “Nigeria and neonate or neonates” from 1990 across three Internet search engines—PubMed, Google Scholar, and the Web of Science.

### Inclusion and exclusion criteria

**Figure 2:**
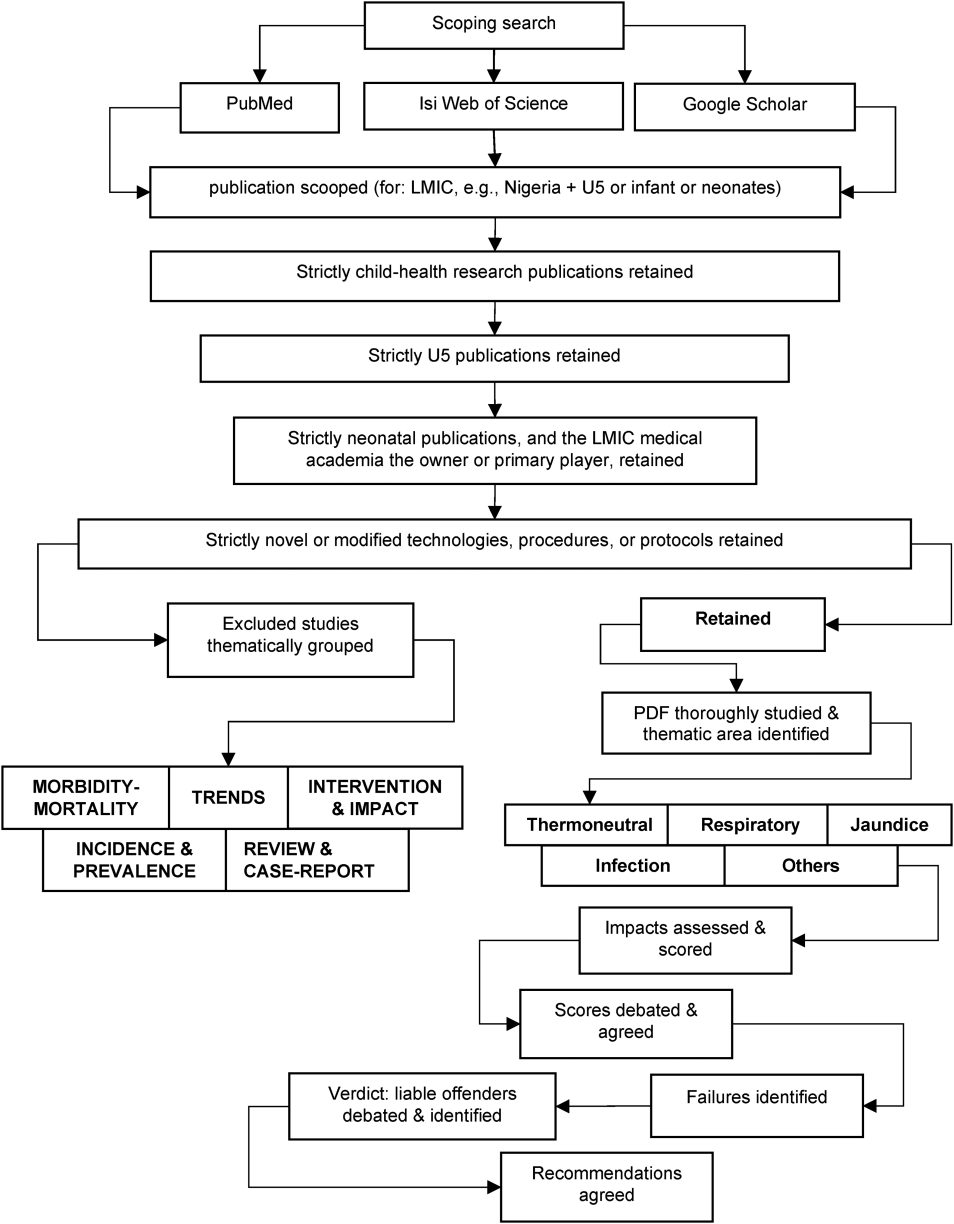
Modified PRISMA for article elimination.

Old and recent publications about novel U5 devices, improved protocols, and modified procedures aimed at improving outcomes, which could have been capable of national scale-up across Nigeria, were identified. All the extracted titles and abstracts of these publications were imported into Rayyan Systematic Review software (11). In brief, Rayyan is an Internet-based systematic review platform that enables team members of a study to independently access the same workspace to assess, exclude, or include extracted titles. In Rayyan, the team leader uploads all publications for assessment and turns-on a “blindfold” key to ensure that each team member conducts their assessments privately without seeing others decisions until every member has finished their review. The leader schedules an online meeting for assessment reconciliation when the “blindfold” key is turned off to reveal how the team members judged the publications and to possibly debate “conflicting” judgements, which are those publications that failed unanimous “inclusion” or “exclusion” by all team members. We designed six stages of the rigorous technique to eliminate non-qualifying articles per stage (Figure 2). A fresh Rayyan environment was initiated and blinded for the jurors' independent assessments and judgements in each stage. Article rejection criteria for the stages were the following:

(1) non-paediatrics publications;
(2) not strictly related to U5 patients or research;
(3) not neonate-specific, not Nigeria-specific, not published by academics in Nigeria, or anchored by a Nigerian researcher for cases of authorship involving non-Nigerians;
(4) not primarily about new or modified devices, improvement protocols, or procedures for better outcomes—jurors were required to choose the reasons for exclusion from a dropdown menu;
(5) final elimination stage—the portable document format (PDF) of accepted publications were uploaded to the Rayyan stage 5 portal to aid full understanding of its contents and to re-assess paper's eligibility as strictly “novel” or “modified”—“novel” refers to previously non-existent devices for solving existing problems, while “modified” pertains to existing techniques systematically improved for better outcomes.

The “included” publications were re-grouped based on topical issues they addressed.

(6) Jurors extracted information relating to the technique's subsequent success rate, national coverage, or impacts, awarding assessment scores, as given in Table 1. Jurors carried out this function through independent wider literature search and direct contact and interviews with available co-authors of the technique.

**Table 1:** Potentially game-changing intervention ideas.

Group	S No.	Article id	Idea presented with its describing article(s)	Current development status (fully developed and available Y/N)	Known user coverage (Y/N)			Reported success rate on 600—900 g and sinkhole neonates (score: 0–10)	Nationwide user coverage	Why poor coverage or whose failing? (1 – 6)
					>1 SCBU? If “Y,” how many are known?	>1 Nigeria region? If “Y,” how many are known?	>1 State? If “Y,” how many are known?			
(A) Neonatal thermoneutral support ideas	1.	Amadi et al. 2014, EFS	A weatherproof nursery design (12)	Y	13	3	7	8	14/74	3, 4, 5
	2.	Amadi et al. 2007, RIT	Digitally recycled incubators (13)	Y	24	3	21	9.5	24/74	3, 4, 5
	3.	Ige et al. 2021, Incubator hood	Suitability of hood geometry (14)	N	N	N	N	N/A	N/A	1, 3
	4.	Amadi 2012, Handy approach (HHA)	Neonatal thermoneutrality in a tropical climate (15)	Y	>24	3	21	9.7	24/74	3, 4, 5
	5.	Amadi et al. 2017, HISA	A novel technique that minimises early neonatal mortality (16)	Y	>8	3	>9	9.8	9/74	3, 4, 5
(B) Neonatal respiratory support ideas	1.	Obu et al. 2020, PoliteO_2_ blend	Air-oxygen blender for respiratory support (17, 18)	Y	2	1	2	8.5	2/74	1, 3, 4, 5, 6
	2.	Onwe et al. 2020, The PSS	A novel oxygen-splitter system (19, 20)	Y	4	2	4	10	4/74	1, 3, 4, 5
	3.	Amadi et al. 2019, Polite-heart CPAP	A low-cost commercial bubble CPAP machine (21)	Y	10	3	6	9.3	10/74	1, 3, 4, 5
	4.	Audu et al. 2013, ibCPAP	Customised bubble CPAP device (22, 23)	Y	47	3	37	0	47/74	1, 3, 4, 5
(C) Neonatal jaundice intervention ideas	1.	Powell et al. 2020, Irradiance meter	A smartphone-enabled phototherapy meter (24)	Y	N	N	N	N/A	N/A	1, 5, 6
	2.	Olusanya et al. 2017, Nomogram	Transcutaneous bilirubin nomograms in African neonates (25)	Y	3	2	3	8	3/74	1, 3
	3.	Slusher et al. 2013, Filtered sunlight	Treatment of neonatal jaundice with filtered sunlight (26)	Y	N/A	2	2	N/A	N/A	1, 4, 5, 6
	4.	Abdulkadir et al., Phototherapy	Nigerian-fabricated phototherapy devices (27)	Y	10	3	6	5	10/74	1, 4, 5, 6
(D) Other topical issues	1.	Amadi et al. 2022, NRS	A community-integrated concept (2)	Y	4	3	4	8	4/74	3, 4, 5
	2.	Modekwe et al. 2021, Oral ketamine	Trial on the efficacy and safety of oral ketamine in circumcision (28)	Y	N	N	N	N/A	1/74	1, 3, 4, 5
	3.	Sobowale et al. 2020, Fuzzy system	A clinical decision support system-based neonatal monitor (29)	Y	2	N	N	N/A	2/74	1, 3
	4.	Emuoyibofarhe et al. 2019, Fuzzy logic	A fuzzy rule-based model for remote monitoring of preterms (30)	N	N	N	N	N/A	0/74	1
	5.	Eregie et al. 1991, GA estimation	A simplified method of estimating gestational age (31)	Y	3	2	3	6	3/74	1, 3, 4, 5
	6.	Amadi and Abubakar 2023, Polite-Light-Bank	LMIC facility-lighting technology (32)	Y	4	2	3	9	4/74	4, 5

Nationwide user coverage (in fraction): It is based on an assumed total of 74 tertiary SCBUs in Nigeria, justified via literature evidence or by referenceable observation and facility contacts. Why poor coverage or who is to blame—(1) tool, (2) neonate, (3) medical academia, (4) hospital management, (5) federal and state ministries of health, and (6) foreign partners.

EFS, evening fever syndrome; RIT, recycled incubator technology; HHA, handy approach; HISA, initial setpoint algorithm; PSS, politeO_2_ splitter system; CPAP, continuous positive airway pressure; ibCPAP, improvised bubble CPAP.

### Impacts and outcomes assessed

Jurors were to take notice of the beneficiary population—whether the technology was used in one facility or across multiple centres, spanning one or more climatic regions (southern, middlebelt, and northern), and whether its usage extended across one or more of the states of Nigeria. The success rate of a technology was evaluated based on its effectiveness in addressing the weakest point of the neonatal life spectrum—the sinkhole region—represented by birthweights of 600–900 g during the F7D period (Figure 1). Scores were graded as follows: 0–2 for no impacts, 3–6 for low impacts, and 7–10 for high impacts. The measurement criterion was strictly based on published, referenceable data demonstrating successful treatment outcomes for a fraction of *n* >9 “sinkhole neonates” or referenceable quantitative data from any of the Nigerian tertiary hospitals. Sinkhole neonates were adjudged “successful” with the applied piece of technology or life-support protocol if the application was proven to have delivered the expected positive outcome towards neonates' eventual survival. The nationwide usage score was determined as the fraction of the total referral special care baby units (SCBUs) in Nigeria adopting the technology. Assuming an average of two tertiary SCBUs per Nigerian state, full nationwide coverage was assumed at 74.

### Jury sittings

The jurors, the arbiters, and the observers assembled virtually, all logging into the Rayyan environment stage being discussed. The chief arbiter initiated the meeting and disabled the “blindfold” key, allowing each juror to see how others judged the elimination criterion. All the publications that were unanimously included or excluded by all four jurors (paediatricians) automatically moved into the Rayyan “include” or “exclude” file lists, respectively. All the remaining articles automatically moved into the Rayyan “conflicted” judgement list. The “conflicted list” tool identified all articles selected by three of the four jurors, which were then also moved to the “include” file. All the publications accepted by only one juror were moved to the “exclude” file. Publications accepted by only two jurors were brought forward for joint reassessment by the jurors, presided over by the chief arbiter. After dialogue on the article in question, the four jurors voted on its inclusion or exclusion. In the event of a tie, the assistant arbiter cast the deciding vote. All jury sittings were conducted via Zoom.com Online Conferencing (Zoom Video Communications, Inc., San Jose, CA, United States) and WhatsApp conference calls, simultaneously. The combined use of these two communication platforms enabled us to combat the limitations posed by poor Internet connectivity for jurors joining from Nigeria. Any conflict of interest relating to any jury member resulted in their recusal until a decision was reached in their absence.

The assessment outcome guided the jurors to deliberate and agree on verdicts on the failures of the NMA, if any, and their co-defendants. The jurors agreed on recommendations on how the co-defendants might encourage, inspire, or influence researchers to dwell more on game-changing studies that could have eliminated the high “sinkhole” NMR.

## Results

**Figure 3:**
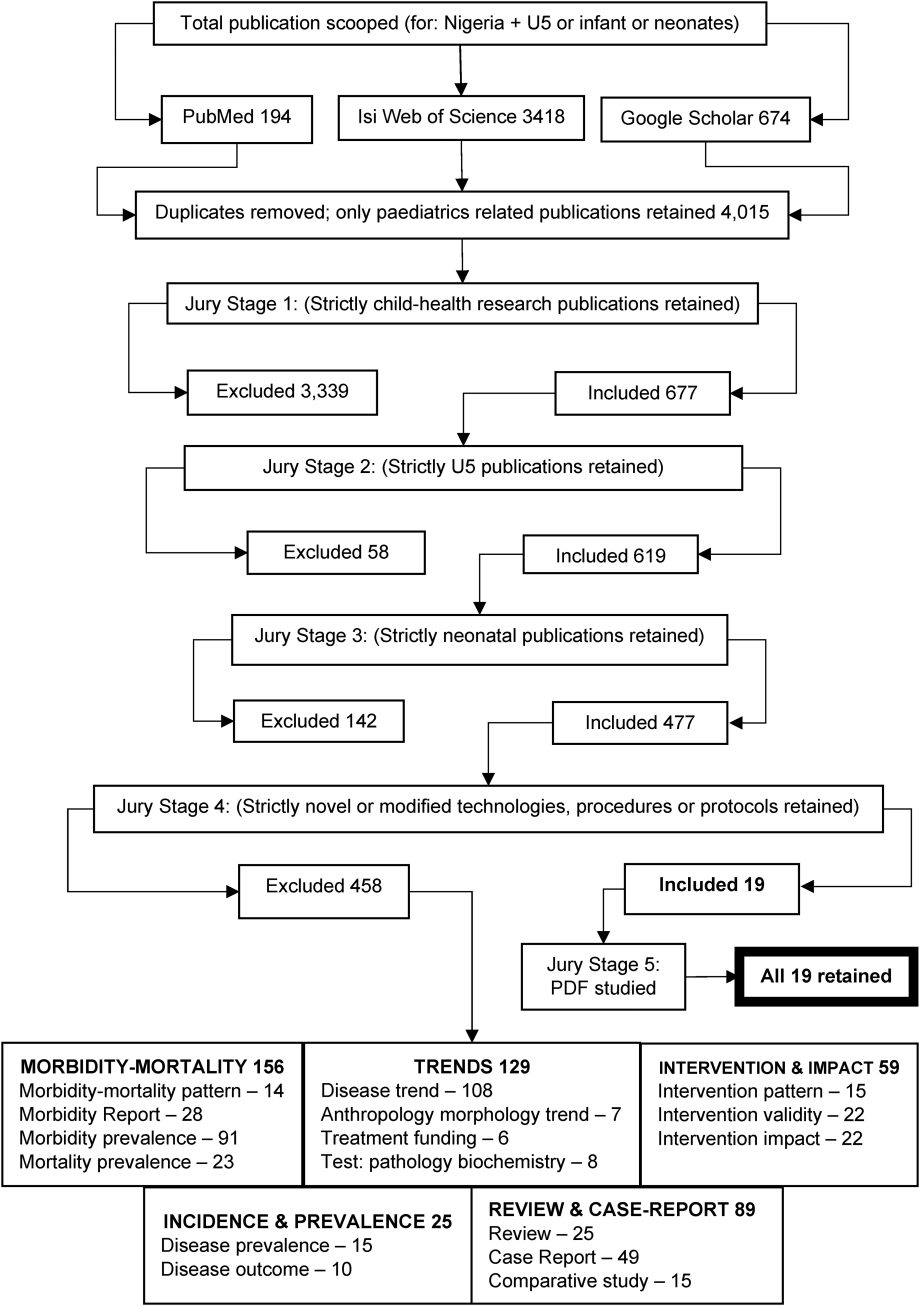
Inclusion and exclusion summary chart.

The search engine scoping pooled 194 publications from PubMed, 673 from Google Scholar, and 3,418 from Web of Science, producing a total of 4,286 articles. The removal of duplicates left 4,015 articles for assessment. The stage-wise elimination process left only 19 pieces of intervention techniques, as shown in Figure 3 (2, 12–17, 19, 21–31). The stage 1 filtration exercise was completed after a cumulative of 39 individual juror working sessions and a total of 1,162 h. Stage 2 lasted 45 sessions and totalled 1,254 h, stage 3 lasted 38 sessions and totalled 1,149 h, stage 4 lasted 29 sessions and totalled 796 h, and stages 5 and 6 involved tens of sessions and thousands of hours, excluding jury sitting hours.

Some of the 19 ideas produced impressive results during their trials and subsequent usage at a few tertiary hospitals. However, none of these applications gained full national coverage, hence failing to scale up nationally. The academia and solution creators were unable to ensure wider usage of their successful ideas. Most reviewed papers demonstrated no evidence of agency funding or other support from the FMOH or hospital management. There was no evidence of adoption or encouragement by the FMOH for these potential game changers. The full assessment of the 19 potentially game-changing ideas is presented in Table 1—thematic areas as identified included the following: (1) five papers regarding thermoneutral support, (2) four focused on respiratory support, (3) four on the diagnosis and management of jaundice, and (4) six additional interventions. Notable amongst them were “Treatment of neonatal jaundice with filtered sunlight” (26), “A novel oxygen-splitter system that expands the utility of oxygen cylinder by up to 700%” (19, 20), “A new low-cost commercial bubble CPAP machine” (21), and “A novel air-oxygen blender for neonatal respiratory support,” which was fully described in a more recent publication (18).

## Discussion

Critical concerns, such as the trend of persistent high neonatal mortality rate in LMICs, are resolved by the intervention of indigenous local scientists, amongst other players, who understand the health situation and possess a personal patriotic passion for ending the suffering of their people. Frugal usage of available time and resources for making a significant impact must, therefore, target identified research questions and gaps, focusing on those with the highest likelihood of contributing to overall mortality reduction. Any other strategy that ignores the weightier gaps could be described as “misfiring,” and could go on for many years without changing the overall situation. Game-changing solutions must necessarily be created around bridging the weightier gaps, without which the overall situation remains unsolved. It could therefore be concluded that the unacceptably high neonatal mortality that has lasted for more than 32 years in Nigeria is an issue for which its weightier gaps are either undiscovered or left without a deliberate synthesis of problem-specific interventions.

We aimed to investigate whether the NMA and associated agencies (the co-defendants) have contributed to the age-long high death rate of the Nigerian neonate (the plaintiff) by failing to identify the weightier gaps and develop effective problem-specific interventions for these. It is understood that importing foreign-developed devices and ideas is a quicker option for LMICs to bridge these gaps. There is nothing wrong with this approach, as research is, by definition, international. However, LMIC researchers are certainly responsible for conducting high-quality intervention science/operational research studies to assess effectiveness, labouring to tweak ideas or creating their own culturally compliant versions. However, they often fail to appreciate that technology importation alone may not sustainably solve the problem without knowledgeable tweaking of associated issues of operational infrastructure, culture, and climate. Therefore, there may be no shortcut to avoiding “getting dirty on the research bench” for LMIC medical academia.

Previous publications by the WHO provided insights into the real gaps fuelling the high U5 mortality rate in Nigeria. By carefully piecing together these publications, it was possible to unravel where research efforts should have been channelled to enhance overall reduction in U5 deaths in Nigeria (Figure 1). Hence, the FMOH and NMA needed to (1) identify successful research that has implemented solutions that are likely to be successful in the Nigerian context of healthcare provision, particularly targeting F7D preterm and low-birth-weight neonates—the so-called “sinkhole” stage of the neonatal life spectrum, (2) promote and strategically scale up these solutions to reach neonates even in most remote areas of Nigeria, and (3) encourage more locally driven research to improve the existing solutions by ensuring that deployable research funds for U5 interventions, whether from the Nigerian Government or any partners, are allocated based on the fraction of the total mortality burden represented by neonatal deaths. The first clue from the WHO implies that noticeable progress could be achieved by allocating 50% of the available funding to neonatal care (4). The second clue suggests that 80% of neonatal deaths occurred during F7D (5). Therefore, to make noticeable progress, 80% of neonatal funding and energies should focus on research targeting the first quarter of neonatal life. From the third clue, we found that >75% of deceased F7D neonates had low birth weight and/or were born prematurely (8). Therefore, good progress could have made by channelling 75% of deployable funding towards research activities targeting the weaker neonates of age F7D. However, these resource-allocation strategies were never implemented.

However, after a total of 141 individual juror assessment sessions covering 4,361 h and numerous gruelling jury sittings, jurors found only 19 out of 4,015 publications from the NMA containing potentially game-changing innovations. Jurors considered this outcome of only 19 potentially game-changing ideas over 32 years to be insufficient; hence, they agreed that, despite their hard work, the NMA did not demonstrate enough leadership or play a sufficient role in synthesising adequate solutions for the aching problems. Furthermore, there was no evidence that these few innovations were encouraged, patronised, or scaled up by the co-defendants in this case. It is noteworthy that in high-income countries, discoveries often do not lead to rapid implementation, as the process may take numerous years. However, none of the assessed innovations in this inquest received any specific funding or assistance to support indigenous developers in reaching neonates in remote areas of Nigeria. Hence, the victims suffered considerably through preventable deaths from the time these innovations were discovered. We consider this failure as a negation of responsibilities by the NMA, FMOH, and hospital management. Some blame also falls apportioned on all support agencies, which for many years have not insisted on proportionately allocating funds to weightier gaps. The jury identified weaknesses and culpabilities across many sides, including the failure of the academia to raise concerns on problem areas for research, failures by implementers despite knowing what has been discovered to work, and the failure of the government to fund/support these efforts. Global research funders could have made exclusive calls for research to address this specific problem (neonatal sinkhole) in LMICs but failed to do so.

### Verdict

With no prejudice prior to this investigation, the jurors have carefully examined all evidence from extracted publications and unanimously agreed to uphold that the Nigerian neonate has not been given a fair chance of survival in the last 32 years by the defendants and co-defendants and, hence, wish to state the following:

The jury unanimously agreed to hold that the so-called sinkhole of neonatal life is the most devastating but unchallenged healthcare gap that has kept NMR high in Nigeria. The jury primarily faults the Nigerian healthcare system, represented by the FMOH and the NMA, which has failed to apply and disseminate information on available novel technologies and innovations within the country. The implementers of global health policies in Nigeria also failed the LMIC neonates—and every group is held responsible for their failure to act or ask the right questions when it became obvious that the anticipated results were not being realised. The actions of the FMOH and the academia in the last 32 years did not demonstrate a full knowledge of the devastating “sinkhole” as explained in this inquest. Therefore, the devastating F7D was left unchallenged till date. Funding grants were not made available by the Nigerian Government or indicative research advocacy policy by the FMOH, who should be responsible for developing innovative policies, monitoring the adherence to the policies, and ensuring that the right researchers received the right amount of support and inspiration. Foreign partners should not be held responsible very much because Nigeria needed to have gotten her acts right and insisted on the best course of action for her problems. The jury apportions part of the fault on the tool developers who could have done better in marketing the developed ideas by striking a fair balance between “money making” and “life saving.” Nigeria is always mentioned internationally as an important country, and there are Nigerian representatives in all the UN bodies—they should have worked harder, made more noise for the Nigerian neonates, and requested more information and data from the home academia to be able to correctly represent Nigeria and what it needs. They all failed the neonates.

### Recommendations

Novel medical devices that have been created and adapted for the Nigerian climate for premature and/or low-birth-weight neonates, which have undergone clinical trialling with published significant success rates but with low nationwide usage coverage, such as those mentioned in this report, should all be brought to the attention of policymakers and stakeholders as ideas deserving of promotion and adoption to enhance neonatal interventions in remote and rural hinterlands across Nigeria.

The concept of a community-integrated neonatal rescue scheme (NRS) in resource-poor environments is brilliant and embraces intervention at three key levels of neonatal care (2). Upon its introductory publication in 2022, its third level of care (the hub centre) already demonstrated huge success in Minna metropolis (Niger State), reducing neonatal mortality from 90% to 4% in 6 years; this is one scheme that could be launched nationwide as soon as possible and serve as a good lesson for the other LMICs (33). Algorithms such as the handy approach (HA), as described in the IntechOpen publication (15), and the initial set-point algorithm (ISA) (16) are validated tools that have demonstrated evidence-based success rates (34) and should be scaled up in caregiving.

Research aimed at diagnosing and managing preventable life-threatening complications in neonates and improving neonatal outcomes of “sinkhole”-classified neonates should be at the forefront of efforts by the LMIC academia. Strategic ideas, concepts, designs, and proposals proven effective by research should be fast-tracked or adapted where needed and scaled up by the appropriate committees and bodies assigned to these roles.

## Data Availability

The raw data supporting the conclusions of this article will be made available by the authors without undue reservation.
